# The potential protein marker of bipolar disorder

**DOI:** 10.1192/j.eurpsy.2022.417

**Published:** 2022-09-01

**Authors:** A. Seregin, E. Dmitrieva, G. Simutkin, S. Ivanova

**Affiliations:** 1 Tomsk National Research Medical Center of the Russian Academy of Sciences, Mental Health Research Institute, Tomsk, Russian Federation; 2 Mental Health Research Institute, Tomsk National Research Medical Center of the Russian Academy of Sciences, Laboratory Of Molecular Genetics And Biochemistry, Tomsk, Russian Federation

**Keywords:** bipolar disorder, proteomics, biomarker

## Abstract

**Introduction:**

Difficulties in the diagnosis of bipolar disorder (BD) are associated with a lack of understanding of the mechanisms of its pathogenesis. Identification of proteins involved in the pathogenesis of BD will bring us closer to an understanding of its mechanisms and can help in diagnosis.

**Objectives:**

The search of proteomic biomarkers of bipolar disorder.

**Methods:**

We performed a proteomic analysis of the serum of 16 healthy people and 33 patients with BD. Patients were hospitalized in an acute state of the depressive phase, and they did not receive therapy for more than 6 months. Blood was collected before the start of therapy. Serum was purified from major proteins by affinity chromatographyandseparatedby1D-electrophoresis. After trypsinolysis, the proteins were identified by HPLC/mass spectrometry. The ELISA kit was used to determine the amount of zNMDAR1.

**Results:**

We identified a protein that does not occur in healthy people: a subunit of the glutamate NMDA receptor zeta-1 (zNMDAR1). As a result, we found a statistically significant (p = 0.037) almost fivefold increase in the concentration of this protein in the serum of patients with bipolar disorder (0.64 [0.18; 0.78] ng/ml) compared with healthy individuals.

**Conclusions:**

Thus, in bipolar disorder NMDAR is damaged, which can lead appearance of their subunits in the serum, and which indicated a violation of glutamatergic neurotransmission. Then this protein claims the role of markers of bipolar disorder. *Mass spectrometric analysis was carried out of the “Human Proteome” Core Facility of the Institute of Biomedical Chemistry Moscow. RSW project, state registration number AAAA-A19-119020690013-2.*

**Disclosure:**

No significant relationships.

